# Integrating HIV care and treatment into tuberculosis clinics in Lusaka, Zambia: results from a before-after quasi-experimental study

**DOI:** 10.1186/s12879-018-3392-2

**Published:** 2018-10-26

**Authors:** Michael E Herce, Jill Morse, Dora Luhanga, Jennifer Harris, Helene J Smith, Stable Besa, Graham Samungole, Nzali Kancheya, Monde Muyoyeta, Stewart E Reid

**Affiliations:** 1 Centre for Infectious Disease Research in Zambia (CIDRZ), Lusaka, Zambia; 2 Division of Infectious Diseases, Department of Medicine, University of North Carolina School of Medicine, Chapel Hill, North Carolina USA; 3 Division of Infectious Diseases, Department of Medicine, University of Alabama at Birmingham School of Medicine, Birmingham, AL USA; 4 Lusaka District Health Office, Ministry of Health, Government of the Republic of Zambia, Lusaka, Zambia

**Keywords:** TB-HIV integration, Tuberculosis, HIV/AIDS, Zambia, Linkage to care, Antiretroviral therapy, Sub-Saharan Africa

## Abstract

**Background:**

Patients with HIV-associated tuberculosis (TB) often have their TB and HIV managed in separate vertical programs that offer care for each disease with little coordination. Such “siloed” approaches are associated with diagnostic and treatment delays, which contribute to unnecessary morbidity and mortality. To improve TB/HIV care coordination and early ART initiation, we integrated HIV care and treatment into two busy TB clinics in Zambia. We report here the effects of our intervention on outcomes of linkage to HIV care, early ART uptake, and TB treatment success for patients with HIV-associated TB in Lusaka, Zambia.

**Methods:**

We provided integrated HIV treatment and care using a “one-stop shop” model intervention. All new or relapse HIV-positive TB patients were offered immediate HIV program enrolment and ART within 8 weeks of anti-TB therapy (ATT) initiation. We used a quasi-experimental design, review of routine program data, and survival analysis and logistic regression methods to estimate study outcomes before (June 1, 2010—January 31, 2011) and after (August 1, 2011—March 31, 2012) our intervention among 473 patients with HIV-associated TB categorized into pre- (*n* = 248) and post-intervention (*n* = 225) cohorts.

**Results:**

Patients in the pre- and post-intervention cohorts were mostly male (60.1% and 52.9%, respectively) and young (median age: 33 years). In time-to-event analyses, a significantly higher proportion of patients in the post-intervention cohort linked to HIV care by 4 weeks post-ATT initiation (53.9% vs. 43.4%, *p* = 0.03), with median time to care linkage being 59 and 25 days in the pre- and post-intervention cohorts, respectively. In Cox proportional hazard modelling, patients receiving the integration intervention started ART by 8 weeks post-ATT at 1.33 times the rate (HR = 1.33, 95% CI: 1.00–1.77) as patients pre-intervention. In logistic regression modelling, patients receiving the intervention were 2.02 times (95% CI: 1.11–3.67) as likely to have a successful TB treatment outcome as patients not receiving the intervention.

**Conclusions:**

Integrating HIV treatment and care services into routine TB clinics using a one-stop shop model increased linkage to HIV care, rates of early ART initiation, and TB treatment success among patients with HIV-associated TB in Lusaka, Zambia.

## Background

Dual tuberculosis (TB) and HIV epidemics have disproportionately affected sub-Saharan Africa (SSA). In 2016, an estimated 30% of all incident TB in the World Health Organization (WHO) African region, or approximately 764,000 total TB cases, were HIV-associated [1]. In Zambia, one of the 30 WHO high TB burden countries, 58% of patients with new or relapse tuberculosis have HIV infection [1]. In Zambia and throughout SSA, patients with HIV-associated TB often have their TB and HIV managed independently, typically in disease-specific vertical care programs [2, 3].

Historically, these vertical programs have provided care for each disease with little to no coordination, relying instead upon separate clinics and different health workers [4]. As a result, these “siloed” programs are associated with diagnostic and treatment delays for both diseases, poor HIV counselling and testing (HCT) uptake, delays in, and low rates of, linkage to HIV care and antiretroviral therapy (ART) initiation, and increased patient morbidity and mortality [5–8] These non-integrated approaches have also been hampered by several operational challenges, including: a lack of resource sharing between TB and HIV programs; uncoordinated visit scheduling, which increases patient costs and work absenteeism; and poor communication between TB and HIV clinics about prescribed drug regimens, treatment-related side effects, and drug-drug interactions for shared patients [3, 9–11]. Importantly, the vertical approach also threatens TB infection control efforts when co-infected patients with newly diagnosed or undiagnosed TB congregate with other HIV-positive patients in ART clinics [12].

In response to these challenges, several models of coordinated TB/HIV care have emerged, reflecting a spectrum of service integration. These models range from referral-based approaches in which co-infected patients are directed from the TB clinic to a separate ART clinic, and vice versa, for TB/HIV services to a more integrated “one-stop shop” model where one healthcare team provides fully integrated TB/HIV services under one roof [3, 11]. Integrated TB/HIV care, in which the same healthcare team provides services to patients with HIV-associated TB, offers potential advantages over standard approaches. First, it allows clinicians to better co-manage TB/HIV co-infection and attendant complications, such as drug-drug interactions, side effects, and toxicities. Second, integrated care helps counsellors and treatment supporters provide psychosocial support and adherence counselling to co-infected patients in the same location. Finally, integrated TB/HIV services helps patients better access treatment and care, including ART, in a timely fashion, without having to wait in multiple clinics within one or more health facilities [3].

For ART provision, clinical trial evidence clearly demonstrates the importance of starting ART early, within 8 to 12 weeks, to reduce HIV-associated TB mortality, particularly for patients with advanced immunosuppression [13–16]. Despite the known benefits, however, several barriers to early ART initiation in patients with HIV-associated TB have hampered uptake of this evidence-based intervention in routine care settings. These barriers include poor coordination between vertical TB and HIV programs, patient and provider reluctance, delays in laboratory testing and result reporting, and the excessive number of steps patients must navigate along the HIV and TB care cascades [5, 9, 17, 18].

In Zambia, while recent progress has been made in improving the proportion of HIV-positive TB patients on ART reported to WHO, from 60% in 2012 to 83% in 2016 [1, 19], challenges remain. For example, in 2016, ART coverage as a percentage of the estimated number of new HIV-associated TB cases nationally stood at just under 60%, suggesting a need for continued work to improve access to ART for HIV-positive TB patients [1]. For HIV-positive TB patients accessing ART, little is known about the average time to ART initiation in routine care settings in SSA. Furthermore, there is limited data from Zambia and the region evaluating integrated TB/HIV care models in real-world clinical settings, and, in particular, few reports examining models that provide comprehensive HIV services, including ART initiation and follow-up, *within* TB clinics.

To increase the proportion of patients with HIV-associated TB who link to HIV care and initiate ART early in routine TB clinical settings, we piloted integration of HIV care and treatment—including HIV care enrolment and ART initiation and follow-up—into two busy TB clinics in Lusaka using a one-stop shop model. The Centre for Infectious Disease Research in Zambia (CIDRZ) implemented the pilot in partnership with the Lusaka District Health Office (DHO) of the Zambian Ministry of Health (MOH). We aim here to assess the feasibility of our pilot intervention and to evaluate its effects on timely linkage to HIV care, early ART uptake, and TB treatment outcomes among HIV-associated TB patients in Lusaka, Zambia.

## Methods

### Study design, population, and setting

We conducted a quasi-experimental before-after study from June 1, 2010 through March 31, 2012 to assess whether our TB/HIV treatment and care integration intervention had an effect on two primary outcomes: linkage to HIV care and early ART initiation. We defined “linkage to HIV care” as documented evidence of having enrolled in the national HIV program. We defined “early” ART initiation as a patient receiving ART within 8 to 12 weeks of anti-TB therapy (ATT) start. Patients were eligible for study inclusion if they had a recorded ATT start date documented in the TB register, and had documented HIV infection. We excluded patients who: were already on ART at the time of TB diagnosis; transferred into a study site TB clinic from a non-study site; were receiving HIV care through a private clinic or non-governmental organization; started ATT outside the defined pre- and post-intervention time periods; or were concurrently enrolled in another study. No patients with documented multi-drug resistant TB received treatment at primary health centre level during the study period.

We observed outcomes of interest among patients meeting study eligibility criteria at two TB clinics situated within two CIDRZ-supported primary health centres in Lusaka, Zambia—“Clinic A” and “Clinic B”. Observations occurred during an 8-month pre-intervention period (June 1, 2010 through January 31, 2011) and an 8-month post-intervention period (August 1, 2011 to March 31, 2012). Patients who started ATT during the pre-intervention period were considered part of a “pre-intervention cohort”; those who initiated ATT during the post-intervention period were part of the “post-intervention cohort”. The pre- and post-intervention periods were exactly the same duration, and were specified to enable an intervening 1-month “wash-out” window between when the last patient joining the pre-intervention cohort completed their 6-month ATT course and the start of our intervention during the post-intervention period.

### Standard of care

Patients in the pre-intervention cohort received the standard of care. Under the standard of care, patients with TB/HIV co-infection are provided TB and HIV care separately in two distinct clinics using a referral-driven model. Typically, the standard of care follows one of two clinical pathways (detailed below) depending on whether HIV or TB is diagnosed first: 1) a newly diagnosed or established HIV-positive patient undergoes evaluation and treatment for TB; or 2) a new TB patient tests positive for HIV.

Newly diagnosed HIV-positive patients may be referred to the ART clinic from any of a number of locations in the facility, including the HCT room, the maternal & child health department, the out-patient department, or other department. At the ART clinic, the patient is enrolled in the national HIV program (requiring registration in the SmartCare electronic medical record system), and undergoes baseline clinical and laboratory evaluation in preparation for ART initiation. Those newly establishing HIV care also receive co-trimoxazole preventive therapy (CPT). All newly diagnosed HIV-positive patients undergo TB screening at the time of their first clinical evaluation by sputum smear microscopy, or, less commonly, by Xpert MTB/RIF (Cepheid, Sunnyvale, CA, USA) where available. Chest x-ray is not routinely available in all health facilities, and where available often requires that a user fee be paid. Patients found to be smear-negative are assessed by a clinician and may be treated empirically based on clinical findings suggestive of TB disease. After establishing HIV care, HIV-positive patients are screened for TB-compatible symptoms at every ART clinic visit per national guidelines [20, 21]. HIV-positive patients diagnosed with TB clinically (i.e. based on symptoms, physical exam, and/or chest x-ray) or microbiologically (i.e. by smear microscopy or Xpert MTB/RIF) are referred to the TB clinic to initiate ATT.

All newly diagnosed TB patients are offered HCT at enrolment into TB care, typically within the TB Clinic itself. To that end, the TB clinic usually houses one or more HCT counselling rooms, and is staffed by one to two nurses and peer health educators. Patients testing HIV-positive are referred to the ART Clinic to establish HIV care and undergo further evaluation for ART initiation as detailed above.

All patients with drug-susceptible TB, irrespective of HIV status, receive first-line ATT with a WHO-recommended, 6-month rifampicin-based fixed-dose combination regimen. ATT is initiated in the TB clinic according to national guidelines from the National Tuberculosis & Leprosy Control Programme (NTP). In Zambia, TB treatment is supported by a lay cadre of peer health educators who provide psychosocial support and adherence counselling to patients.

### HIV care and treatment integration intervention

We implemented the same model of TB/HIV service integration in two routine TB clinics—Clinic A and Clinic B. The integration intervention was introduced first at Clinic A in August 2011, and then at Clinic B in October 2011. The intervention had five core components: 1) health worker training and mentorship; 2) timely provider-initiated HIV testing and counselling (PITC); 3) on-site HIV care enrolment; 4) dedicated ART clinic days; and 5) synchronized TB and HIV patient follow-up. We further describe these components below.

We trained all nurses and clinical officers from both the TB clinic and ART clinic on TB/HIV co-management. Training emphasized proper ATT and ART prescribing practices, and recognition and management of drug-related side effects and toxicity. Importantly, clinicians were trained to begin ART for all co-infected patients as soon as possible, and preferably within 8 to 12 weeks of ATT initiation, regardless of CD4 count. CIDRZ clinician mentors worked intensively with facility staff for approximately 6 months at the start of the intervention to reinforce training concepts. CIDRZ peer educators were trained to provide health education talks to patients on the relationship between TB and HIV and the need for early and sustained co-treatment.

**Fig. 1:**
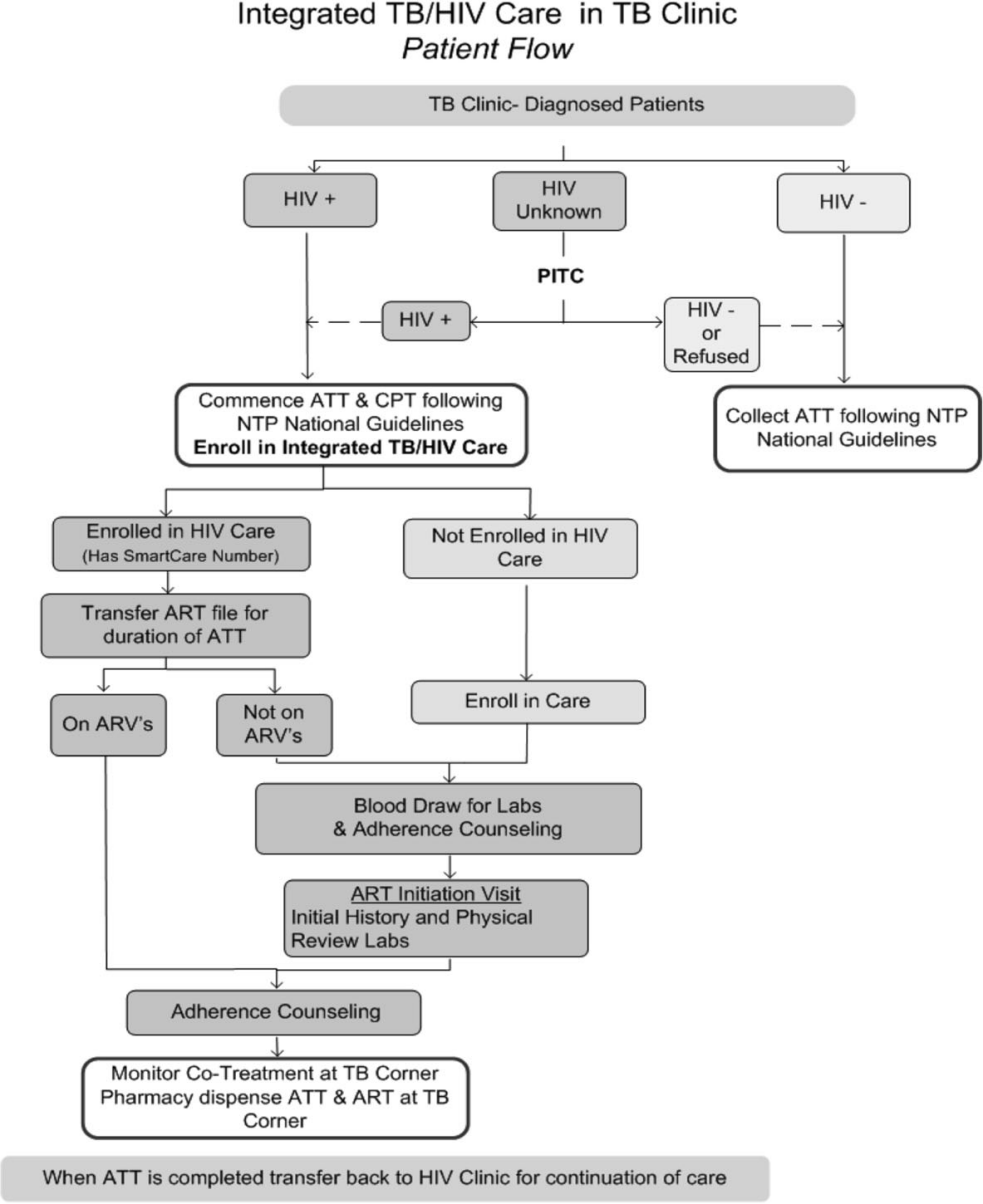
Integrated TB/HIV care patient flow. HIV, human immunodeficiency virus; TB, tuberculosis; PITC, provider-initiated testing and counselling; ATT, anti-tuberculosis therapy; CPT, co-trimoxazole therapy; ART, anti-retroviral therapy; ARVs, antiretrovirals; NTP, National Tuberculosis and Leprosy Programme

All newly diagnosed TB patients were offered PITC at enrolment into TB care (Fig. 1). TB patients newly identified as HIV-positive who had not previously enrolled in HIV care were informed about the importance of linking to HIV care and starting ART, and were offered immediate enrolment into HIV care on site. MOH nursing staff conducted initial HIV care enrolment procedures in line with national guidelines in place at the time of the study, including: completion of the MOH HIV care enrolment form and blood draws for baseline laboratory tests (including complete blood count, CD4, creatinine, and liver function tests). Following HIV care enrolment, patients were scheduled for an on-site ART initiation visit with a clinical officer to undergo a thorough clinical evaluation, including history, physical exam, and laboratory test result review, and to start ART. HIV-positive TB patients already on ART at the time of ATT initiation were given the option to continue their HIV care at the ART clinic or to transfer their HIV care to the TB clinic for the duration of ATT.

To ensure adequate patient follow-up and efficient use of limited human resources for clinician staffing, each TB clinic scheduled a weekly ‘ART clinic day’ when the same MOH clinical officer was posted to provide “one-stop shop” TB/HIV services, including ART initiation. On the scheduled day, the MOH clinical officer evaluated patients, conducted a physical exam, and reviewed lab results and ART eligibility. Once patients enrolled in HIV care, and initiated ART, they attended the TB clinic according to their directly observed therapy (DOT) schedule (daily, weekly or monthly) for TB treatment. ART and TB follow-up was synchronized to harmonize clinical care and drug collection, and to maximize patient convenience. Specifically, the same clinical officer provided follow-up clinical evaluation for both TB and HIV and attendant co-morbidities and drug-related toxicities, and patients collected both their antiretrovirals (ARVs) and TB medications from the same open-air drug dispensary at the TB clinic. Eligible patients who declined to initiate ART continued to receive TB care in the TB clinic and were encouraged to start ART at each visit. Upon ATT completion, patients were referred to the ART clinic in each facility for continuing their HIV care.

### Data collection

For both the pre- and post-intervention periods, data were collected prospectively during routine clinical care, and recorded onto the following primary data sources: national TB registers, patient TB treatment cards, paper-based HIV care files, and the national SmartCare HIV electronic medical record system. From these data sources, we subsequently abstracted patient data fields of interest, including: age, sex, TB clinic serial number, TB registration date, TB case identification number, TB registration type (i.e. new, relapse, transferred in, treatment after default, or failure), TB anatomical site (i.e. pulmonary TB [PTB] or extra-pulmonary TB [EPTB]), TB treatment start date, recorded HIV status, SmartCare registration number, date of HIV care enrolment, date of initial history and physical exam, date of first CPT dispensation, date of first ART dispensation, and WHO TB treatment outcome [22, 23]. Blank data fields were coded as “missing.” Per routine medical record keeping practice, any patient without a documented HIV care enrolment date is considered not to have linked to care, and any patient without a first ART dispensation date is thought not to have started ART. All data were de-identified at the time of abstraction to protect patient confidentiality. For the pre-intervention cohort, we administratively censored HIV care enrolment and ART initiation 6 months after the end of the pre-intervention period, on July 31, 2011. Similarly, for the post-intervention cohort, we censored HIV care enrolment and ART initiation 6 months after the end of the post-intervention period, on September 30, 2012.

### Data analysis

Patient characteristics at the time of ATT initiation (i.e. at “baseline”) were compared between pre- and post-intervention cohorts using the Chi-square test for categorical variables and the Student’s t-test for continuous variables. Simple proportions were used to describe the primary outcomes: 1) the percentage of HIV-associated TB patients newly enrolled in HIV care by 4, 8, and 12 weeks of ATT initiation; and 2) the percentage of HIV-associated TB patients who newly initiated ART by 4, 8, and 12 weeks of starting ATT. We compared the proportion of patients with a primary outcome between the pre- and post-intervention cohorts, stratified by study clinic, using the Cochran-Mantel-Haenszel test. We estimated time to HIV care enrolment and ART initiation using Kaplan-Meier methods with follow-up time measured from ATT initiation. Cumulative failure functions were compared using the Log rank test. Cox proportional hazard modelling was employed to estimate the association between exposure to our TB/HIV integration intervention and ART start by 8 weeks post-ATT initiation. We selected 8 weeks post-ATT initiation as our threshold of interest as this is the WHO-recommended time frame by which ART should be initiated in patients with HIV-associated TB [24]. To estimate the effect of the intervention on successful TB treatment (i.e. TB cure or treatment completion), multi-variable logistic regression modelling was used to determine the odds ratio and associated 95% confidence interval (CI). For both our Cox proportional hazard and logistic regression models, we adjusted for potential confounders that were selected on the basis of clinical and programmatic plausibility, including age, sex, intervention clinic, TB anatomical site, and TB registration type (i.e. new case or cases of relapse, default, or treatment after failure). Patients who transferred out or had missing outcomes data were excluded from the models. We considered two-sided *p*-values ≤0.05 statistically significant. All statistical analyses were performed using STATA version 12.1 (College Station, TX, USA).

### Ethics statement

Ethical approval for this study was granted by the University of Zambia Biomedical Research Ethics Committee and the Institutional Review Board at the University of North Carolina at Chapel Hill, without requiring patient consent given the use of de-identified, routinely collected data.

## Results

### Overview

Over the 22-month study period, Clinic A and B registered 578 and 983 total HIV-positive and HIV-negative TB patients, respectively; HIV prevalence among TB patients at Clinic A and B was 68.3% and 55.5%, respectively. A total of 473 patients met study eligibility criteria, including 248 patients in the pre-intervention cohort and 225 patients in the post-intervention cohort.

### Baseline patient characteristics

Patient age and sex distribution did not differ significantly between the pre- and post-intervention cohorts, with a majority of patients being male with a mean age of 33 years (Table 1). Eighty-five percent of patients in both the pre- and post-intervention cohorts were classified as having new TB. A similar majority of patients in both cohorts had pulmonary tuberculosis, with the remaining patients in both cohorts having extra-pulmonary tuberculosis. While 52.8% of patients in the pre-intervention cohort hailed from Clinic A, 65.8% of patients in the post-intervention cohort were from Clinic B—a statistically significant difference (Table 1). The proportion of HIV-positive TB patients *not* linked to care at baseline as measured by enrolment in the HIV program at the time of ATT initiation was significantly higher in the post-intervention cohort at 74.2%, compared to 64.1% in the pre-intervention cohort (*p* = 0.02) (Table 1).

**Table 1:** Baseline Characteristics of Pre- and Post-Intervention Cohorts

Characteristic	Pre-intervention *N* = 248 n (%)	Post-intervention *N* = 225 n (%)	*p*-value
Sex			
Male	149 (60.1)	119 (52.9)	0.12
Female	99 (39.9)	106 (47.1)	
Age, mean (se)	33.7 (0.7)	33.3 (0.7)	0.74
TB Registration Type			
New	211 (85.1)	192 (85.3)	0.40
Relapse	35 (14.1)	33 (14.7)	
Treatment after Default or Failure	2 (0.8)	0 (0)	
TB Anatomical Site			
Pulmonary TB	207 (83.5)	184 (81.8)	0.63
Extra-Pulmonary TB	41 (16.5)	41 (18.2)	
Clinic			
Clinic A	131 (52.8)	77 (34.2)	< 0.01
Clinic B	117 (47.2)	148 (65.8)	
Linkage to care Status			
Not Linked *(Not Enrolled in HIV Care, not receiving ART)*	159 (64.1)	167 (74.2)	0.02
Linked *(Enrolled in HIV Care, not receiving ART)*	89 (35.9)	58 (25.8)	

*se* Standard error, *TB* Tuberculosis, *ART* Antiretroviral therapy

### Linkage to HIV care

A total of 147 patients had documented evidence of HIV program enrolment at the time of ATT initiation, and thus were excluded from the linkage to care analysis. Among patients who were not linked at baseline, a significantly higher proportion of patients in the post-intervention cohort linked to HIV care by 4, 8, and 12 weeks after ATT initiation, compared to patients in the pre-intervention cohort at the same time points (Table 2). By 12 weeks after ATT initiation, 62.9% of patients in the post-intervention cohort had linked to HIV care, compared to 54.7% in the pre-intervention cohort (*p* = 0.03). Stratified by clinic, we observed a significantly higher proportion of patients in the post-intervention cohort at Clinic A linking to care by 4, 8, and 12 weeks. For Clinic B, we did not observe a statistically significant difference in the proportion of patients in the pre- and post-intervention cohorts linking to HIV care by the time points of interest (Table 2).

**Table 2:** Linkage to HIV care outcomes for patients not already in HIV care at baseline (*N* = 326)

Outcome	Pre-intervention	Post-intervention	*p*-value
Linked to HIV care within 4 weeks of ATT initiation			
All patients	69/159 (43.4%)	90/167 (53.9%)	0.01
Clinic A	40/81 (49.4%)	41/55 (74.6%)	< 0.01
Clinic B	29/78 (37.2%)	49/112 (43.8%)	0.37
Linked to HIV care within 8 weeks of ATT initiation			
All patients	82/159 (51.6%)	99/167 (59.3%)	0.04
Clinic A	46/81 (56.8%)	45/55 (81.8%)	< 0.01
Clinic B	36/78 (46.2%)	54/112 (48.2%)	0.78
Linked to HIV care within 12 weeks of ATT initiation			
All patients	87/159 (54.7%)	105/167 (62.9%)	0.03
Clinic A	48/81 (59.3%)	47/55 (85.5%)	< 0.01
Clinic B	39/78 (50.0%)	58/112 (51.8%)	0.81

*ATT* Anti-TB therapy

**Fig. 2:**
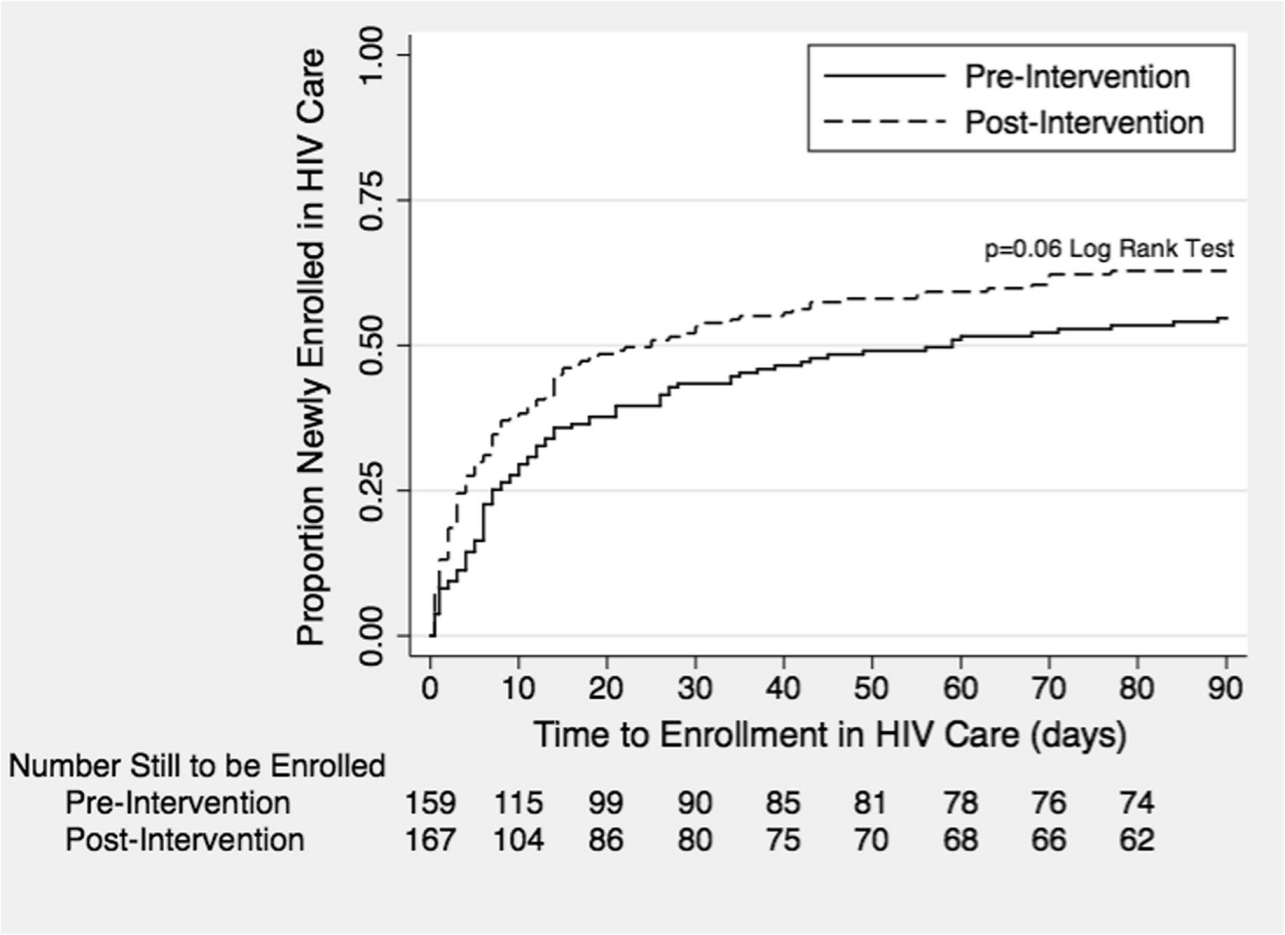
Time to HIV care linkage in the pre- and post-intervention cohorts

In time-to-event analyses, a significantly higher proportion of patients in the post-intervention cohort compared to the pre-intervention cohort linked to care by 4 weeks (53.9% vs. 43.4%, *p* = 0.03), but not by 8 weeks (59.3% vs. 51.6%, *p* = 0.07) or 12 weeks (62.9% vs. 54.7%, *p* = 0.06) (Fig. 2). The median time to HIV care linkage was 59 days in the pre-intervention cohort versus 25 days in the post-intervention cohort.

### ART initiation

We evaluated ART initiation in both patients who were linked and not linked to HIV care at baseline (Table 3). The proportion of all patients at Clinics A and B initiating ART in the post-intervention cohort by 4 weeks after ATT initiation was higher in the post-intervention cohort (29.3%) than the pre-intervention cohort (25.0%), however, this difference was not statistically significant (*p* = 0.18) (Table 3). A significantly higher proportion of patients initiated ART by 8 weeks in the post-intervention (45.3%) versus pre-intervention cohort (38.3%) (*p* = 0.04). Similarly, by 12 weeks after ATT, significantly more HIV-positive TB patients receiving the intervention had started ART (52.0%) than those who had not been exposed to the intervention (41.5%) (*p* < 0.01). The proportion of patients starting ART within 4, 8, and 12 weeks of ATT initiation was observed to be consistently higher in Clinic A than Clinic B for both the pre- and post-intervention cohorts.

**Table 3:** ART initiation outcomes for patients not yet on ART at the time of TB treatment initiation (*N* = 473)

Outcome	Pre-intervention	Post-intervention	*p*-value
Initiating ART within 0 to 4 weeks			
All patients	62/248 (25.0%)	66/225 (29.3%)	0.18
Clinic A	40/131 (30.5%)	23/77 (29.9%)	0.92
Clinic B	22/117 (18.8%)	43/148 (29.1%)	0.05
Initiating ART within 0 to 8 weeks			
All patients	95/248 (38.3%)	102/225 (45.3%)	0.04
Clinic A	58/131 (44.3%)	41/77 (53.3%)	0.21
Clinic B	37/117 (31.6%)	61/148 (41.2%)	0.11
Initiating ART within 0 to 12 weeks			
All patients	103/248 (41.5%)	117/225 (52.0%)	< 0.01
Clinic A	61/131 (46.6%)	51/77 (66.2%)	< 0.01
Clinic B	42/117 (35.9%)	66/148 (44.6%)	0.15

*ART* Antiretroviral therapy

**Fig. 3:**
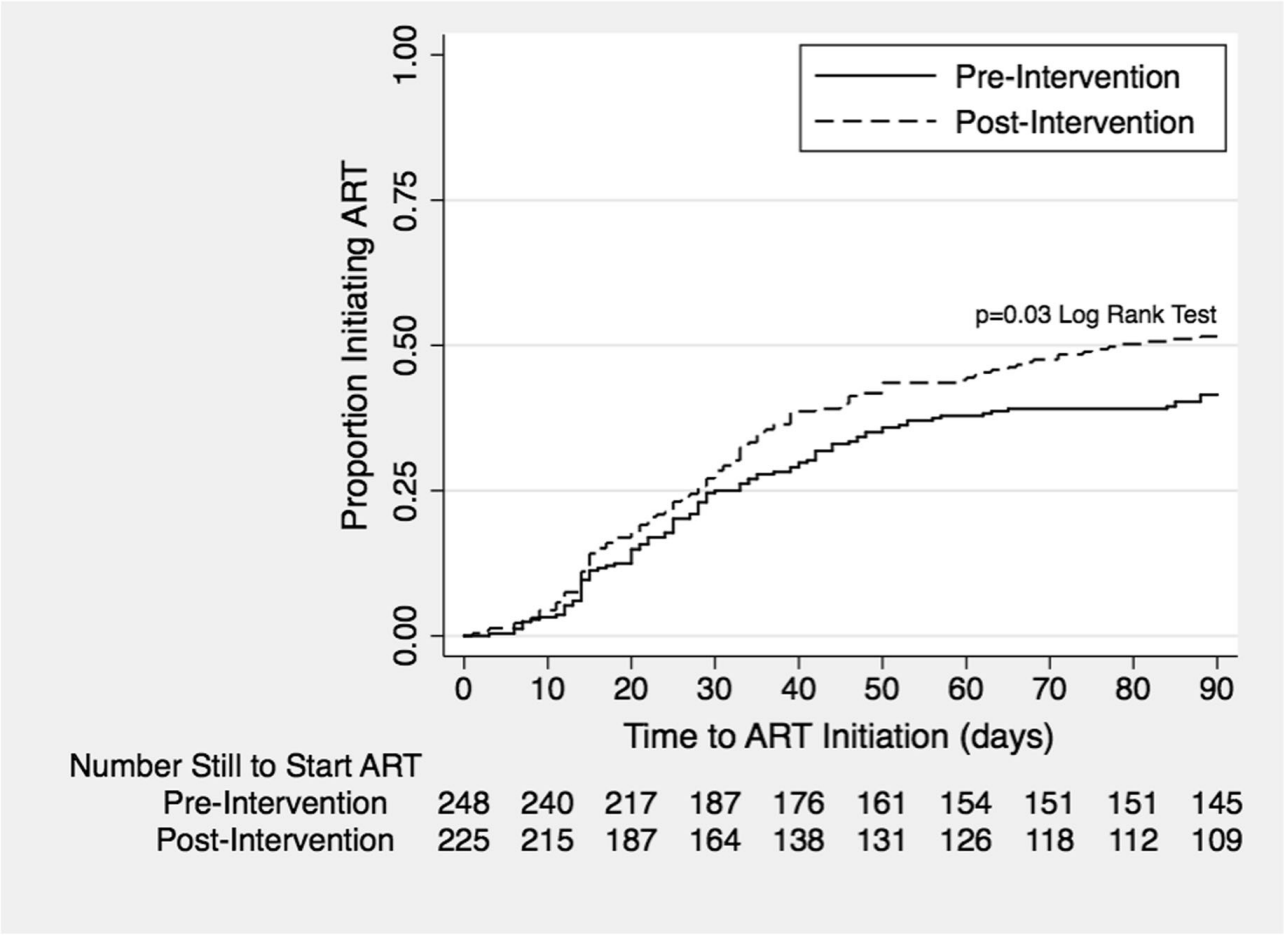
Time to ART in the pre- and post-intervention cohorts (censored at 12 weeks)

In time-to-event analyses, a significantly higher proportion of patients in the post-intervention versus pre-intervention cohort had initiated ART early, by 12 weeks (52.0% vs. 41.5%, *p* = 0.03) after TB treatment initiation (Fig. 3), but not by 8 weeks (45.3% vs. 38.3%, *p* = 0.11) or 4 weeks (29.3% vs. 25.0%, *p* = 0.28). Overall, median time to ART was 264 days in the pre-intervention cohort and 78 days in the post-intervention cohort.

In our adjusted multi-variable Cox proportional hazard model controlling for age, sex, clinic, TB anatomical site, and TB registration type, TB patients who received the integration intervention started ART by 8 weeks post-ATT at 1.33 times the rate (HR = 1.33, 95% CI: 1.00–1.77) as patients who were not exposed to the intervention, and this observation was statistically significant (Table 4).

**Table 4:** Estimated effect of the integrated TB/HIV treatment and care intervention, and other covariates, on ART initiation by 8 weeks (N = 473)

Characteristic	Unadjusted		*p*-value	Adjusted^a^		*p*-value
	HR	95% CI		HR	95% CI	
Integration Intervention						
After	1.25	(0.95, 1.66)	0.12	1.33	(1.00, 1.77)	0.05
Before	1.00					
Sex						
Female	0.82	(0.62, 1.09)	0.18	0.84	(0.63, 1.12)	0.23
Male	1.00					
Age						
≥ 35 years	1.21	(0.91, 1.60)	0.19	1.17	(0.89, 1.56)	0.26
< 35 years	1.00					
Clinic						
Clinic B	0.73	(0.55, 0.97)	0.03	0.71	(0.53, 0.94)	0.02
Clinic A	1.00					
TB Site						
Extra-pulmonary	0.79	(0.53, 1.17)	0.24	0.81	(0.55, 1.20)	0.29
Pulmonary	1.00					
TB Registration Type^b^						
Treatment after Default, Failure, OR Relapse	0.89	(0.60, 1.33)	0.57	0.84	(0.56, 1.26)	0.40
New	1.00					

*HR* Hazard ratio, *TB* Tuberculosis

^a^HR estimated by Cox proportional hazard modelling, adjusting for remaining variables presented in the table

^b^Relapse, treatment after default, and failure were collapsed into one category due to collinearity in the model

### TB treatment outcomes

Among the 473 total patients with HIV-associated TB in the pre- and post-intervention cohorts, 284 (60.0%) had a documented TB treatment outcome. Of these, a higher proportion had a successful outcome (i.e. either microbiological cure or documented treatment completion) in the post-intervention cohort (83.3%) compared to the pre-intervention cohort (73.9%), although the difference was of borderline statistical significance (*p* = 0.051) and the effect was less pronounced in Clinic B than Clinic A (Table 5).

**Table 5:** Proportion of patients who either were cured or completed TB treatment during the pre- and post-intervention periods (*N* = 284)

Population	Pre-intervention *N* = 134 n (%)	Post-intervention *N* = 151 n (%)	*p*-value
All patients	99/134 (73.9%)	125/150 (83.3%)	0.05
Clinic A	60/77 (77.9%)	53/58 (91.4%)	0.04
Clinic B	39/57 (68.4%)	72/92 (78.3%)	0.18

In adjusted multivariable logistic regression modelling, patients receiving the TB/HIV integration intervention were 2.02 times (95% CI: 1.11–3.67) as likely to have a successful TB treatment outcome (i.e. achieving cure or completing treatment) as patients not receiving the intervention (Table 6).

**Table 6:** Estimated effect of the integrated TB/HIV treatment and care intervention, and other covariates, on successful TB treatment (cure or completion) (N = 284)

Characteristic	Unadjusted		*p*-value	Adjusted^a^		*p*-value
	OR	95% CI		OR	95% CI	
Integration Intervention						
After	1.77	(0.99, 3.15)	0.05	2.02	(1.11, 3.67)	0.02
Before	1.00					
Sex						
Female	1.10	(0.61, 1.98)	0.75	1.16	(0.64, 2.13)	0.62
Male	1.00					
Age						
≥ 35 years	1.05	(0.59, 1.87)	0.87	1.08	(0.60, 1.95)	0.81
< 35 years	1.00					
Clinic						
Clinic B	0.57	(0.32, 1.02)	0.06	0.50	(0.27, 0.92)	0.03
Clinic A	1.00					
TB Site						
Extra-pulmonary	0.77	(0.35, 1.69)	0.52	0.82	(0.37, 1.82)	0.62
Pulmonary	1.00					
TB Registration Type^b^						
Treatment after Default, Failure OR Relapse	0.94	(0.42, 2.10)	0.89	0.99	(0.43, 2.28)	0.98
New	1.00					
ART started within 8 weeks of TB treatment						
Yes	1.30	(0.72, 2.32)	0.38	1.16	(0.63, 2.13)	0.63
No	1.00					

*OR* Odds ratio

^a^OR estimated by logistic regression modelling, adjusting for remaining variables presented in the table

^b^Relapse, treatment after default, and failure were collapsed into one category due to collinearity in the model

## Discussion

We demonstrate that implementing a one-stop shop model of TB/HIV service integration significantly increases linkage to HIV care and rates of early ART initiation among patients with HIV-associated TB. Providing this integrated model in TB clinics does not detract from TB services, but rather appears to increase the odds of TB cure or treatment completion. Taken together, our findings suggest the feasibility of integrating HIV care and treatment within routine TB clinical settings in Lusaka, Zambia.

Despite increasing access and coverage of ART in Zambia [25], there is an on-going need to implement and evaluate integrated TB/HIV service delivery models to ensure universal access to ART for patients with HIV-associated TB. Challenges with linkage to HIV care and ART initiation for HIV-positive TB patients persist despite clear national guidelines calling for prompt ART initiation and compelling clinical trial data documenting the clear benefits of early ART [13, 26–28]. Reasons for sub-optimal linkage to HIV care and ART uptake among patients with HIV-associated TB are thought to be multifactorial, and include: increased patient transport and opportunity costs related to the multiple visits generated by referral-based services; fear among patients and clinicians about the co-management of side effects and toxicities; the greater pill burden resulting from co-treatment; and stigma associated with establishing HIV care and initiating ART [6, 9, 11, 29–31].

WHO guidelines strongly recommend that ART be started within 8 weeks of ATT initiation [24]. Zambian national TB and HIV guidelines reinforce this guidance, calling for early ART initiation as soon as ATT is tolerated, ideally within 2 to 3 weeks of commencing TB treatment [20]. Yet, no published reports from Zambia describe the extent to which ART is started early, or the average time to ART initiation from ATT initiation, for patients with HIV-associated TB in routine care settings. In our post-intervention cohort, we observed an average time to ART from ATT initiation of 78 days, a marked reduction from the 264 days observed in the pre-intervention cohort. Similarly, patients who received our intervention had an increased rate of early ART initiation (by 8 weeks), although ART uptake remained below 50%. While these findings suggest our intervention improved access to early ART, the relatively low absolute proportion of patients who received ART by 8 weeks argues for greater investments in HIV services and health system strengthening to improve integrated TB/HIV care for patients with HIV-associated TB. In our Lusaka study clinics, we postulate that the relatively low ART uptake observed by 8 and 12 weeks was due to persistent gaps in human resources for health, clinical infrastructure in TB practice settings, and other service delivery barriers.

Our intervention featured a one-stop shop model characterized by complete integration of HIV treatment and care within existing TB clinics. Using this model, HIV-infected TB patients received PITC, CPT, ART and TB services in one clinic space under the care of the same providers who managed both TB and HIV at the same visit. Opt-out PITC may have been particularly important for linking co-infected patients to HIV care as it has been shown previously to result in higher testing uptake than traditional opt-in approaches across a variety of settings [32, 33]. In addition, offering on-site HIV care enrolment and co-localized treatment in the TB clinics minimized patient referral within and between facilities, which we postulate helped reduce the kind of pre-ART loss to follow-up associated with more traditional service integration models [6, 11, 34]. Despite the availability of these services, however, we observed only modest linkage to HIV care by 12 weeks post-ATT initiation, which suggests the influence of other patient-, health system-, and structural-level factors that likely went unaddressed by our intervention.

Although single-facility integration models have been described previously, few reports characterize one-stop shop approaches to integrate ART provision and HIV care *within* TB clinics, and even fewer compare the effects of such approaches using data from historical or experimental controls [11, 29, 35, 36]. In Kenya, integrating HIV treatment and care into a rural TB clinic resulted in an increase in ART initiation (by the end of ATT), from 13% to between 29 and 36% [37]. In South Africa, integrated TB/HIV care was associated with significantly fewer unfavourable ART outcomes, which included death, default, and stopped treatment, than a vertical care approach [38].

We observed that integrated TB/HIV treatment and care was associated with increased odds of successful TB treatment. However, only 83.3% of patients achieved cure or completed treatment in the post-intervention period. While this proportion compares favourably with national figures reported to WHO for the study period, and TB outcomes for other integrated approaches documented in the region [38], it nonetheless falls below the Global Plan to End TB target of at least 90% of patients experiencing treatment success [19, 39]. Of note, TB treatment success at Clinic A exceeded 90%, but failed to surpass 80% in Clinic B, suggesting differences in the implementation context and barriers to introducing our intervention between TB clinics.

In both TB clinics, we encountered several noteworthy barriers to introducing integrated treatment and care. Limited clinic capacity and human resources, in particular, presented barriers to integrated service delivery, as reported elsewhere [11, 40]. At one study clinic, we encountered human resource challenges of frequent staff shortages, scheduling issues, and turnover, and addressed these directly through training, re-training, and mentorship. While it is possible that we could have strengthened our model by placing long-term project staff in the TB clinics, given our resource limitations we opted for a more sustainable approach focused on building the capacity of existing MOH staff to deliver integrated services. Similar to other settings, clinicians reported concerns over managing co-infected patients and co-administering ART and ATT, fearing the development of adverse events, such as immune reconstitution inflammatory syndrome [41, 42]. Some staff were not motivated to support integrated activities, especially if the activities were perceived as extra work. Indeed, overburdened staff has been cited as an important barrier to implementing integrated TB/HIV activities [43]. Anecdotally, we perceived this to be a clinic-specific issue: in Clinic B, reluctance among some staff to adopt the intervention may have contributed to poorer ART initiation outcomes; while in Clinic A, staff more uniformly welcomed TB/HIV integration and acknowledged it as a more efficient way to provide care. Results from other pilot projects suggest that TB/HIV integration may be a means to improve health worker knowledge, motivation, and retention [3]. Lastly, issues with supply chain management in the TB clinic resulted in intermittent sputum container and TB diagnostic reagent stock outs. In Malawi, similar supply chain challenges affected uptake of integrated HIV services among co-infected patients [9].

In responding to these challenges, several “key lessons learned” emerged. First, involving policy makers, facility in-charges, health workers, and other leadership in program planning from the outset was essential for obtaining local buy-in, and highlight the importance of obtaining visible MOH support and sensitizing staff pre-implementation to achieve adequate integration. Second, weekly clinic team meetings provided a forum to discuss challenges and identify and disseminate clinic-level best practices. Third, site-based mentoring enabled clinical officers to develop practical clinical skills and confidence in TB/HIV co-management. Fourth, longitudinal technical support for organizational and logistical problem solving led to improvements in patient flow and supply chain management for TB and HIV commodities. Fifth, adequate clinical space was essential for ensuring ART integration [40]. We found that clinics with at least 3 rooms worked best: one room allowed new patient enrolments and daily medication dispensing; a second room enabled patient examination on ART/TB clinic days; and a third room facilitated health commodity storage and private counselling space for HCT. Sixth, we found that integrated care functioned best when a dedicated, ART-trained clinical officer was assigned to staff the TB clinic. In Zambia, this clinical officer provided necessary clinical support to two nurses overseeing TB clinic operations. These nurses functioned daily to enrol into HIV care any TB patient newly diagnosed with HIV and to schedule appointments for clinician review and ART initiation.

From the client perspective, several patients anecdotally expressed a preference for our integrated care model. Similar patient preference has been reported previously in South Africa, especially for patients from lower socioeconomic strata [44]. Patients expressed an appreciation for the shorter waiting times and reduced stigma in the integrated TB clinic compared to the ART clinic, as well as the added convenience of our one-stop shop approach. In some cases, patients were reluctant to return to their general ART clinic after completion of TB treatment [11]. Qualitative research is required to further examine patient preferences regarding, and the patient-specific challenges surrounding, integrated TB/HIV care [31].

While the absence of qualitative data limited our ability to formally explore patient preferences regarding integrating HIV treatment and care into TB clinics, such an analysis was beyond the scope of this study. Our study was also limited by missing TB treatment outcome data for approximately 40% of the patients in our study. Missing data resulted from the inability to ascertain paper-based TB treatment outcomes for patients who did not return for a final TB clinic visit and could not be traced, and those who transferred out from our study sites during the course of their anti-TB therapy. These issues, along with our study having been conducted in only two urban Zambian clinics, limit the generalizability of our findings. Our study was also not designed to ascertain retention in HIV care, including retention after patient completion of ATT when they were referred to the ART clinic for continued HIV management. Similarly, we did not assess the effects of other important clinical, behavioural, and structural factors on linkage to care and ART uptake for patients with HIV-associated TB, including baseline CD4 count, perceived HIV- and TB-related stigma, co-morbid mental illness and substance use, and distance from patients’ homes to the health facility. Lastly, the use of existing, de-identified program data precluded our ability to corroborate observational findings from elsewhere in SSA suggesting that integrated TB/HIV care reduces mortality among co-infected patients [45, 46].

## Conclusions

We demonstrate that it is feasible to integrate HIV treatment and care, including ART, within public TB clinics in urban Zambia, while concurrently improving TB treatment outcomes. We describe an integrated model of TB/HIV treatment and care characterized by: the same health workers providing both TB and HIV services with the help of longitudinal training and mentorship; dedicated staffing, space, and clinic days for delivering all integrated services in the same clinic; and synchronized TB and HIV follow-up to ensure one-stop shopping for patients seeking care at a single TB clinic. Using this model, we achieved increases in linkage to care and early ART uptake for patients with HIV-associated TB. Additional studies are needed to identify new strategies to further improve integrated care and ART uptake, retention, and viral suppression among patients with HIV-associated TB in Zambia, in line with national and international targets. Further research is also needed to elucidate the patient-, health system-, and structural-level factors that enable or impede integrated care and the impact these factors have on joint, longitudinal TB/HIV clinical outcomes. Finally, additional investments are needed to ensure adequate human resources for health and health systems sufficiently robust to deliver high-quality, integrated services along the entire TB/HIV care continuum for patients with HIV-associated TB in SSA.

## Abbreviations

ART: Antiretroviral Therapy
ARV: Antiretroviral
ATT: Anti-TB therapy
CIDRZ: Centre for Infectious Disease Research in Zambia
CPT: Co-trimoxazole Preventive Therapy
DOT: Directly observed therapy
EPTB: Extra-pulmonary Tuberculosis
HCT: HIV Counselling and Testing
HIV: Human Immunodeficiency Virus
HR: Hazard Ratio
MOH: Ministry of Health
OR: Odds Ratio
PLHIV: People Living with HIV
PTB: Pulmonary Tuberculosis
SSA: Sub-Saharan Africa
TB: Tuberculosis
WHO: World Health Organization

## References

[1] WHO (2017). Global Tuberculosis Report.

[2] Friedland G, Harries A, Coetzee D (2007). Implementation issues in tuberculosis/HIV program collaboration and integration: 3 case studies. J Infect Dis.

[3] Uyei J, Coetzee D, Macinko J, Guttmacher S (2011). Integrated delivery of HIV and tuberculosis services in sub-Saharan Africa: a systematic review. Lancet Infect Dis.

[4] Tsiouris SJ, Gandhi NR, El-Sadr WM, Gerald F (2007). Tuberculosis and HIV-needed: a new paradigm for the control and Management of Linked Epidemics. J Int AIDS Soc.

[5] Lawn SD, Campbell L, Kaplan R, Little F, Morrow C, Wood R (2011). Ie DEASA: delays in starting antiretroviral therapy in patients with HIV-associated tuberculosis accessing non-integrated clinical services in a south African township. BMC Infect Dis.

[6] Zachariah R, Harries AD, Manzi M, Gomani P, Teck R, Phillips M, Firmenich P (2006). Acceptance of anti-retroviral therapy among patients infected with HIV and tuberculosis in rural Malawi is low and associated with cost of transport. PLoS One.

[7] Mukadi YD, Maher D, Harries A (2001). Tuberculosis case fatality rates in high HIV prevalence populations in sub-Saharan Africa. AIDS (London, England).

[8] Zachariah R, Spielmann MP, Chinji C, Gomani P, Arendt V, Hargreaves NJ, Salaniponi FM, Harries AD (2003). Voluntary counselling, HIV testing and adjunctive cotrimoxazole reduces mortality in tuberculosis patients in Thyolo, Malawi. AIDS (London, England).

[9] Kumwenda M, Tom S, Chan AK, Mwinjiwa E, Sodhi S, Joshua M, van Lettow M (2011). Reasons for accepting or refusing HIV services among tuberculosis patients at a TB-HIV integration clinic in Malawi. Int J Tuberc Lung Dis.

[10] Chileshe M, Bond VA (2010). Barriers and outcomes: TB patients co-infected with HIV accessing antiretroviral therapy in rural Zambia. AIDS Care.

[11] Legido-Quigley H, Montgomery CM, Khan P, Atun R, Fakoya A, Getahun H, Grant AD (2013). Integrating tuberculosis and HIV services in low- and middle-income countries: a systematic review. Trop Med Int Health.

[12] Maher D (2010). Re-thinking global health sector efforts for HIV and tuberculosis epidemic control: promoting integration of programme activities within a strengthened health system. BMC Public Health.

[13] Abdool Karim SS, Naidoo K, Grobler A, Padayatchi N, Baxter C, Gray AL, Gengiah T, Gengiah S, Naidoo A, Jithoo N (2011). Integration of antiretroviral therapy with tuberculosis treatment. N Engl J Med.

[14] Havlir DV, Kendall MA, Ive P, Kumwenda J, Swindells S, Qasba SS, Luetkemeyer AF, Hogg E, Rooney JF, Wu X (2011). Timing of antiretroviral therapy for HIV-1 infection and tuberculosis. N Engl J Med.

[15] Grinsztejn B, Hosseinipour MC, Ribaudo HJ, Swindells S, Eron J, Chen YQ, Wang L, Ou S-S, Anderson M, McCauley M (2014). Effects of early versus delayed initiation of antiretroviral treatment on clinical outcomes of HIV-1 infection: results from the phase 3 HPTN 052 randomised controlled trial. Lancet Infect Dis.

[16] Nahid P, Dorman SE, Alipanah N, Barry PM, Brozek JL, Cattamanchi A, Chaisson LH, Chaisson RE, Daley CL, Grzemska M (2016). Official American Thoracic Society/Centers for Disease Control and Prevention/Infectious Diseases Society of America clinical practice guidelines: treatment of drug-susceptible tuberculosis. Clin Infect Dis.

[17] Harris JB, Hatwiinda SM, Randels KM, Chi BH, Kancheya NG, Jham MA, Samungole KV, Tambatamba BC, Cantrell RA, Levy JW (2008). Early lessons from the integration of tuberculosis and HIV services in primary care centers in Lusaka, Zambia. Int J Tuberc Lung Dis.

[18] Howard AA, El-Sadr WM (2010). Integration of tuberculosis and HIV services in sub-Saharan Africa: lessons learned. Clin Infect Dis.

[19] WHO. Global Tuberculosis Report. Geneva: World Health Organization; 2013. p. 289.

[20] Ministry of Health (2016). Zambia Consolidated Guidelines for Treatment & Prevention of HIV Infection.

[21] Ministry of Health (2014). Managing Tuberculosis in the HIV setting in Zambia.

[22] World Health Organization (2017). Guidelines for treatment of drug-susceptible tuberculosis and patient care: 2017 update.

[23] WHO. Treatment of tuberculosis guidelines, 4th Edition, vol. 2010. Geneva: World Health Organization; 2010. p. 147.

[24] WHO. Consolidated Guidelines on the Use of Antiretroviral Drugs for Treating and Preventing HIV Infection: Recommendations for a Public Health Approach. Second ed. Geneva: World Health Organization; 2016. p. 429. 27466667

[25] Ministry of Health. Zambia Population-Based HIV Impact Assessment (ZAMPHIA 2015–2016): Summary Sheet. Lusaka: Ministry of Health, Republic of Zambia; 2016.

[26] Cain LE, Logan R, Robins JM, Sterne JA, Sabin C, Bansi L, Justice A, Goulet J, van Sighem A, de Wolf F (2011). When to initiate combined antiretroviral therapy to reduce mortality and AIDS-defining illness in HIV-infected persons in developed countries: an observational study. Ann Intern Med.

[27] Kitahata MM, Gange SJ, Abraham AG, Merriman B, Saag MS, Justice AC, Hogg RS, Deeks SG, Eron JJ, Brooks JT (2009). Effect of early versus deferred antiretroviral therapy for HIV on survival. N Engl J Med.

[28] Sterne JA, May M, Costagliola D, de Wolf F, Phillips AN, Harris R, Funk MJ, Geskus RB, Gill J, Dabis F (2009). Timing of initiation of antiretroviral therapy in AIDS-free HIV-1-infected patients: a collaborative analysis of 18 HIV cohort studies. Lancet.

[29] Ferroussier O, Dlodlo RA, Capo-Chichi D, Boillot F, Gninafon M, Trébucq A, Fujiwara PI (2013). Integrating HIV testing and care into tuberculosis services in Benin: programmatic aspects [short communication]. Int J Tuberc Lung Dis.

[30] Topp SM, Chipukuma JM, Giganti M, Mwango LK, Chiko LM, Tambatamba-Chapula B, Wamulume CS, Reid S (2010). Strengthening health systems at facility-level: feasibility of integrating antiretroviral therapy into primary health care services in Lusaka, Zambia. PLoS One.

[31] Daftary A, Padayatchi N (2012). Social constraints to TB/HIV healthcare: accounts from coinfected patients in South Africa. AIDS Care.

[32] Van Rie A, Sabue M, Jarrett N, Westreich D, Behets F, Kokolomani J, Bahati ER (2008). Counseling and testing TB patients for HIV: evaluation of three implementation models in Kinshasa, Congo. Int J Tuberc Lung Dis.

[33] Pope DS, Deluca AN, Kali P, Hausler H, Sheard C, Hoosain E, Chaudhary MA, Celentano DD, Chaisson RE (2008). A cluster-randomized trial of provider-initiated (opt-out) HIV counseling and testing of tuberculosis patients in South Africa. J Acquir Immune Defic Syndr.

[34] Wandwalo E, Kapalata N, Tarimo E, Corrigan CB, Morkve O (2004). Collaboration between the national tuberculosis programme and a non governmental organisation in TB/HIV care at a district level: experience from Tanzania. Afr Health Sci.

[35] Phiri S, Khan PY, Grant AD, Gareta D, Tweya H, Kalulu M, Chaweza T, Mbetewa L, Kanyerere H, Weigel R (2011). Integrated tuberculosis and HIV care in a resource-limited setting: experience from the Martin Preuss Centre, Malawi. Trop Med Int Health.

[36] Schwartz AB, Tamuhla N, Steenhoff AP, Nkakana K, Letlhogile R, Chadborn TR, Kestler M, Zetola NM, Ravimohan S, Bisson GP (2013). Outcomes in HIV-infected adults with tuberculosis at clinics with and without co-located HIV clinics in Botswana. Int J Tuberc Lung Dis.

[37] Huerga H, Spillane H, Guerrero W, Odongo A, Varaine F (2010). Impact of introducing human immunodeficiency virus testing, treatment and care in a tuberculosis clinic in rural Kenya. Int J Tuberc Lung Dis.

[38] Schulz SA, Draper HR, Naidoo P (2013). A comparative study of tuberculosis patients initiated on ART and receiving different models of TB-HIV care. Int J Tuberc Lung Dis.

[39] Stop TB Partnership (2015). The Paradigm Shift, 2016-2020: Global plan to end TB.

[40] Martinot A, Van Rie A, Mulangu S, Mbulula M, Jarrett N, Behets F, Bola V, Bahati E (2008). Baseline assessment of collaborative tuberculosis/HIV activities in Kinshasa, the Democratic Republic of Congo. Trop Dr.

[41] Gandhi NR, Moll AP, Lalloo U, Pawinski R, Zeller K, Moodley P, Meyer E, Friedland G (2009). Successful integration of tuberculosis and HIV treatment in rural South Africa: the Sizonq'oba study. J Acquir Immune Defic Syndr.

[42] McIlleron H, Meintjes G, Burman WJ, Maartens G (2007). Complications of antiretroviral therapy in patients with tuberculosis: drug interactions, toxicity, and immune reconstitution inflammatory syndrome. J Infect Dis.

[43] Pope DS, Atkins S, DeLuca AN, Hausler H, Hoosain E, Celentano DD, Chaisson RE (2010). South African TB nurses’ experiences of provider-initiated HIV counseling and testing in the eastern Cape Province: a qualitative study. AIDS Care.

[44] Levin L, Irving K, Dikgang M, Punwasi J, Isaacs M, Myer L (2006). TB patients’ perspectives on integrated TB/HIV services in South Africa. Trop Dr.

[45] Van Rie A, Patel MR, Nana M, Vanden Driessche K, Tabala M, Yotebieng M, Behets F (2014). Integration and task shifting for TB/HIV care and treatment in highly resource-scarce settings: one size may not fit all. J Acquir Immune Defic Syndr.

[46] Kerschberger B, Hilderbrand K, Boulle AM, Coetzee D, Goemaere E, De Azevedo V, Van Cutsem G (2012). The effect of complete integration of HIV and TB services on time to initiation of antiretroviral therapy: a before-after study. PLoS One.

